# An Assessment of Ergonomics Climate and Its Association with Self-Reported Pain, Organizational Performance and Employee Well-Being

**DOI:** 10.3390/ijerph18052610

**Published:** 2021-03-05

**Authors:** Elham Faez, Seyed Abolfazl Zakerian, Kamal Azam, Kyle Hancock, John Rosecrance

**Affiliations:** 1 Department of Occupational Health, School of Public Health, Tehran University of Medical Sciences, Tehran 1417613151, Iran; 2 Department of Epidemiology and Biostatistics, School of Public Health, Tehran University of Medical Sciences, Tehran 1417613151, Iran; Kazam@tums.ac.ir; 3 Department of Environmental and Radiological Health Sciences, Colorado State University, Fort Collins, CO 80523, USA; kyle.hancock@colostate.edu (K.H.); john.rosecrance@colostate.edu (J.R.)

**Keywords:** ergonomics climate, general health, organizational performance, self-reported pain

## Abstract

Previous studies have demonstrated that a positive ergonomics climate with an equal focus on improving operational performance and employee well-being is beneficial to both employee health and organizational performance. This study aimed to assess the ergonomics climate at two power plants and examine its association with self-reported pain, performance, and well-being. At two power plants in Iran, survey responses from 109 and 110 employees were obtained. The questionnaires contained data on ergonomics climate, organizational performance, employee health, and self-reported pain. Results showed that the mean ergonomics climate scores between the Besat and Rey power plants were significantly different (*p* < 0.001). The overall ergonomics climate score, and all subscales scores, were positively associated with organizational performance (*p* < 0.001). The overall ergonomics climate score, and some of its subscales, were significantly associated with employees’ general health (*p* < 0.001). The ergonomics climate score was significantly higher in the group of employees who reported musculoskeletal pain than those who did not report musculoskeletal pain (*p* < 0.05). Investigation of ergonomics climate can provide organizations with a baseline for prioritizing their values and finding areas for improving organizational performance and employee health.

## 1. Introduction

The high level of competition in the global market has compelled companies to implement new technologies, change organizational structure, and introduce novel workplace improvement programs. For example, ergonomics programs with a strong focus on preventing work-related injuries and human error accidents have been employed in various industries [1,2]. Ergonomics is a system-oriented approach focused on both human interactions with work and the design of work processes. Generally, organizations implement ergonomic programs to reduce injury costs, decrease waste, and reduce the rate of absenteeism. Ergonomic programs can also increase employee motivation and productivity while improving the quality of the products and services [3,4,5,6]. Measurements of an organizations ergonomics climate are utilized to quantify the value that an organization places on integrating ergonomics principles to maximize operational performance and well-being outcomes [7]. This measure was first introduced in a study by Hoffmeister et al. [7] at a large manufacturing facility in the United States.

Ergonomics climate was defined as “employee perceptions of the extent to which the organization emphasizes and supports the design and modification of work such that both operational performance and employee well-being are maximized” [7,8]. Climate reflects the employees’ perception and knowledge of the organization’s field of activities and represents the atmosphere and space in which the employees work [9,10]. In the definition of ergonomics climate, operational performance refers to the economic aspects of an organization’s functions. These include productivity, efficiency, quality, sustainability, competitive advantage, and the ability to perform the organization’s task to stay successful [11]. Operational performance is a broad concept that shows the state or quality of performance for different activities and their associated outcomes [12]. Managers often consider these activities a high priority because they directly impact their organization’s productivity and effectiveness [13,14]. As an organization’s human resources, employees play a significant role in improving productivity and effectiveness, which can promote overall organizational performance [15]. Employee well-being refers to the organization’s focus on maintaining a high level of health and safety in the workforce. Some of the variables considered in employee well-being include injury and illness rates, job satisfaction, stress, absenteeism, and work-life balance [16,17,18].

According to the definition of ergonomics climate, the workplace’s design and modification can improve employee well-being while improving operational performance [8]. This approach has long been considered one of the most comprehensive methods for improving the work environment [19,20].

Organizations should strive for a climate that supports both operational performance and employee well-being to maximize their overall success as a company. Organizations that value performance improvement more than health and safety report higher work-related musculoskeletal pain levels among their workforce [21]. Organizations that value employee well-being more than performance may still report higher levels of work-related pain among their employees because of a decline in productivity, increasing pressure to compensate for this lack of productivity in the future. Organizations that equally value performance and employee well-being and act by a system-oriented approach can expect the highest amount of growth and success [7,22]. Although many studies have explored the safety climate [23,24,25] and performance climate [26,27,28], there is only one published study on ergonomics climate in the workplace [7]. Safety climate focuses on employee safety but does not reflect employee work performance, represented by ergonomics climate [29]. Ergonomics climate assesses deeper and more diverse values within an organization than other climate measures. However, like safety climate measures, ergonomic climate measures are also leading, rather than lagging, indicators of work performance outcomes. The purpose of this study was to evaluate ergonomics climate and its association with employee well-being and organizational performance at two Iranian power plants.

## 2. Materials and Methods

### 2.1. Participants

A cross-sectional study was utilized to assess the ergonomics climate at the Besat and Rey power plants in Tehran, Iran. Both plants had similar departments, including maintenance, operations, engineering, planning, and administration. The Rey power plant had previously provided ergonomics training for management and employees. There were 570 active employees total at the two facilities working in different departments. The sample size was estimated using statistical power analysis and the following formula:

(1) $$N=\frac{2*\partial^{2}{\left(z_{1-\frac{\propto }{2}}+z_{1-\beta }\right)}^{2}}{{\left(\mu_{0}-\mu_{1}\right)}^{2}},\;\;\left(\partial^{2}=1.04^{2}\right),$$

whereby power was calculated at 1−β=0.80 with a margin of error *α* = 0.05. According to a previous study, the ***∂*** and μ0−μ1 values were determined as 1.04 and 0.04, respectively. As a result, a minimum of 106 employees was required for recruitment in each power plants. A total of 150 employees were invited to complete the survey in each facility. Employees were randomly selected from different departments at each facility to complete the surveys. Each facility provided a list of all employees, and random numbers were used for employee selection. The response rates were 72% (109 employees) and 73% (110 employees) for the Besat and Rey power plant, respectively. Additional information on the collected sample size is presented in Table 1 and Table 2.

Before data collection, the required information about the study’s purpose and procedures was provided to the employees and their supervisors. Participation in the survey was voluntary, and consent was obtained before participation. The Ethics Committee of Tehran University of Medical Sciences (Project identification code: IR.TUMS.SPH.REC.1396.3728) approved the study’s protocol.

### 2.2. Measures

An Ergonomic Climate Assessment questionnaire [7] was used to measure ergonomics climate score. The Ergonomic Climate questionnaire was translated from English into Persian for the present study by two experts in occupational health and ergonomics, whose native language was Persian. The validity and reliability of the Persian translation were determined in our previous study [30]. A panel of experts composed of 10 professionals in occupational health and ergonomics was assembled to conduct a validity assessment. The panel of experts computed a Content Validity Index (CVI) to determine item relevance and a Content Validity Ratio (CVR) to determine if each item was essential [31,32]. The CVI and CVR were 0.94 and 0.90, respectively. Panel members provided suggestions to improve the content and sentence structure. Reliability was evaluated by using Cronbach’s alpha as a measure of internal consistency. For this purpose, a cross-sectional study was carried out on 50 employees of the Besat power plant. The Cronbach’s alpha was calculated using the SPSS 21 software (*α* = 0.96), which indicate internal consistency reliability based on the George and Mallery guideline [31,33,34]. The Ergonomic Climate measure has consisted of four subscales, including management commitment, employee involvement, hazard identification/control, and training/knowledge. A 5-point Likert scale was used to record the responses according to five possible choices of 1-strongly disagree, 2-disagree, 3-neither agree nor disagree, 4-agree, and 5-strongly agree. The scores were summed for ten subscales, resulting in two operational performance and employee well-being scores. The overall ergonomics climate score was determined by adding the scores of these two values.

Demographic characteristics, including age, gender, and work experience, were collected. The ergonomics climate scores obtained were used to assess the impact of ergonomics climate on self-reported pain, organizational performance, and employee well-being.

Individuals were asked if they had experienced any work-related pain in the past 12 months in nine different areas of their body regarding the measurement of self-reported pain. The yes (1) or no (0) binary variable was used to record self-reported pain. Hersey and Goldsmith Standard Questionnaire and General Health Questionnaire (GHQ) assessed organizational performance and employee well-being. Additional details for all three questionnaires are presented in Table 3.

### 2.3. Data Analysis

The data were analyzed using SPSS-21 upon completion of all questionnaires. Statistical variables were described using parameters including percentage, mean, and standard deviation. The Kolmogorov-Smirnov test and Independent T-test were used to investigate the normality of quantitative variables and analyze the mean difference between the two facilities. The Spearman correlation and Chi-square tests were used to determine the relationship between variables. A *p*-value of 0.05 or less was considered statistically significant [38].

## 3. Results

### 3.1. Descriptive Results

Among the sample population, 95.6% (Besat) and 99.1% (Rey) of the employees were male. Participant’s mean age was 35.2 ± 6.2 and 35.1 ± 6.7 years at Besat and Rey facilities, respectively. Respondents reported work experience in three categories, the largest being in the 5 to 10 years group (53.2% at Besat and 54.6% at Rey). All demographic information on the workers is presented in Table 4.

**Figure 2:**
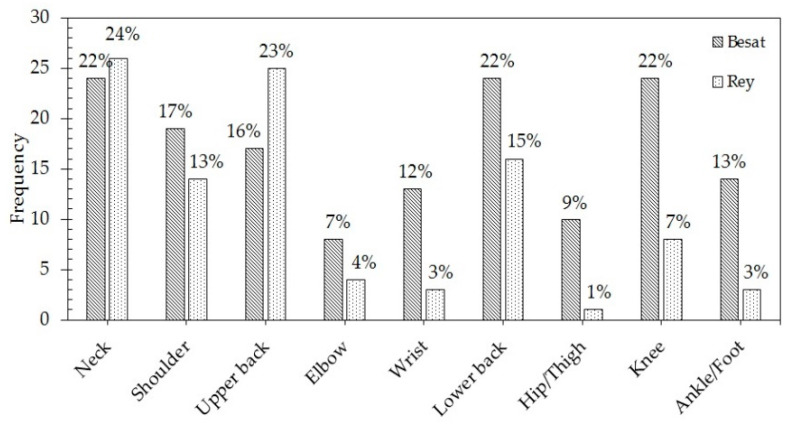
Percentage of self-reported pain by body part from the Ergonomics Climate Questionnaire.

Analysis of the self-reported pain data revealed that employees at the Besat plant experienced the highest level of pain in the neck, lower back, and knee (22%), and employees at the Rey plant experienced the highest level of pain in the neck (23.6%), as depicted in Figure 2.

**Figure 3:**
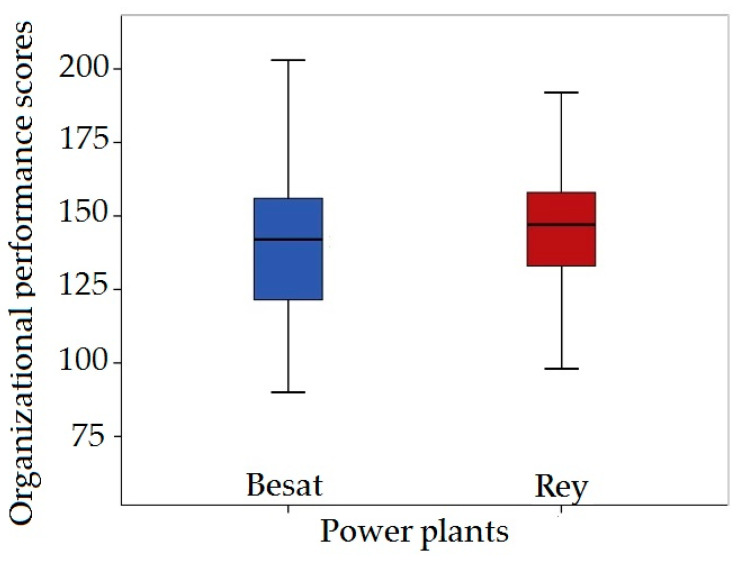
Organizational Performance scores from the Hersey and Goldsmith Questionnaire.

The organizational performance data from the Hersey and Goldsmith Standard Questionnaire indicated that the mean organizational performance was 140.0 ± 23.0 at the Besat plant and 147.7 ± 24.8 at the Rey plant, as shown in Figure 3.

**Figure 4:**
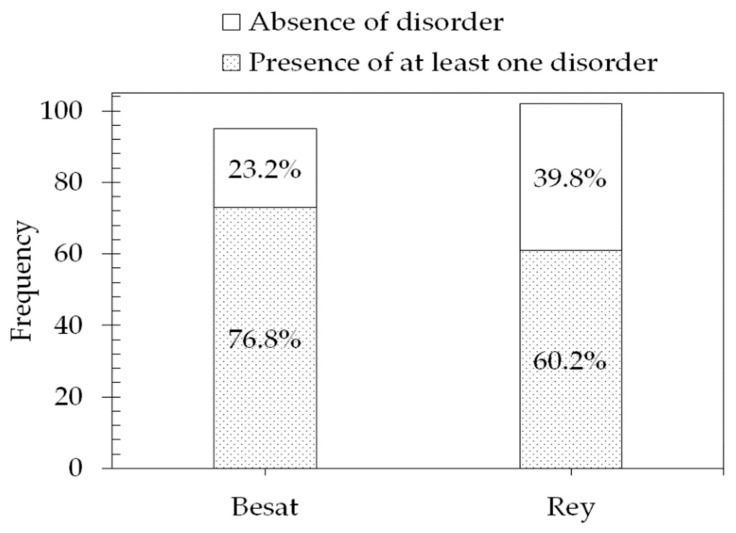
Frequency of disorders from the General Health Questionnaire.

The General Health Questionnaire result indicated that 76.8% and 60.2% of employees at the Besat and Rey plants, respectively, reported the presence of at least one disorder (Figure 4).

### 3.2. Analytical Results

The mean scores of ergonomics climate were significantly different at Besat and Ray power plant (*p* < 0.001). The mean scores of the operational performance and employee well-being facets of the ergonomics climate and their subscales were significantly higher at the Rey facility, as shown in Table 5.

Also, there was a significant difference between the overall ergonomics climate scores of two groups of employees, those who reported at least one general health disorder and those who did not. Those two groups also differed in the subscale scores of management commitment and employee involvement, as shown in Table 6.

There was a significant difference between the mean of overall ergonomics climate scores of the two groups of employees, those who did and did not report musculoskeletal pain in the wrist, lower back, hip/thigh, and ankle/foot Table A1 (Appendix A).

The results of assessing the relationship between two facets of ergonomics climate (i.e., operational performance and employee well-being), as well as their corresponding subscales and self-reported pain in nine areas of the body, are summarized in Table A1 (Appendix A)

**Figure 5:**
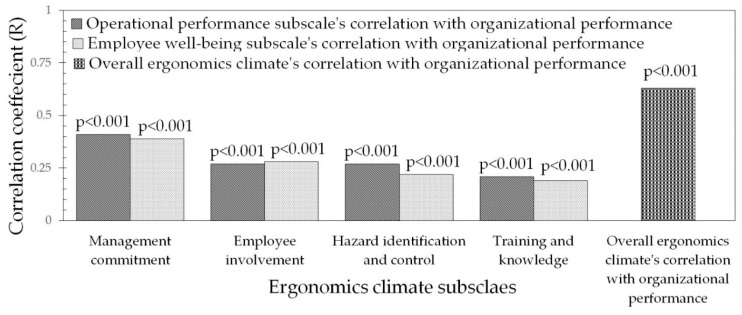
Spearman correlation coefficients for ergonomics climate measures and organizational performance.

A significant correlation was observed between the overall ergonomics climate and each of its subscales with organizational performance. There was a positive and moderate correlation between the overall ergonomics climate and organizational performance (*p* < 0.001), as shown in Figure 5.

## 4. Discussion

This study utilized previously developed measures but is the first to assess the ergonomics climate to investigate its association with employee well-being and organizational performance. In the ergonomics climate subscales, the mean operational performance score was higher than the mean employee well-being score at both facilities.

There was a significant difference between the overall ergonomics climate score and each subscale score at two Rey and Besat power plants. Holding an ergonomics awareness training session for management and employees at Rey power plant may be a primary source for the difference between ergonomics climate scores at the two power plants. In both operational performance and employee well-being, management commitment had the highest mean value at the Rey facility. The difference was significant betweenthe overall ergonomics climate scores and each subscale scores at two power plants. The employee’s perception of management commitment was reported higher at the Rey facility due to various practices. These practices include more collaborative relationships between management and employees, employee involvement in the decision-making to address ergonomics and safety issues, and employees participantion in ergonomics training awareness. Previous studies have introduced management commitment as a factor that can influence the other dimensions of safety [39,40,41].

Further studies indicated the critical role of management commitment in implementing the ergonomic principle and its influence on employee buy-in and commitment to the organization [42,43]. Management commitment to operational performance usually manifests itself in employee training and job enrichment which can ultimately improve employee perceptions regarding the quality of goods and services [44]. Management support and psychosocial attitudes are the most important predictors of an ergonomics program’s success or failure [45].

The ergonomics climate measure included another subscale, employee involvement, which can be positively influenced by management support of employee participation in providing solutions for controlling the workplace hazards. The employee involvment subscale was significantly higher at the Rey plant when compared to the Besat plant. The high level of employee involvement likely influenced the level of perceived ergonomics climate at the Rey facility. Previous studies have found that the employee perception of an organization’s climate directly affects the employee perception of involvement [46,47]. The climate of an organization should be considered an essential factor in promoting employee involvement. Ultimately, a climate of management support and commitment encourages employee involvement, directly influencing climate perception [48,49].

Training and knowledge also promoted a higher perception of the ergonomics climate at the Rey plant. Training employees leads to self-protection practices, resulting in several beneficial outcomes for the organization [50]. Similar to our research finding, Mazzetti et al. (2020) demonstrated that the perception of a safety climate among construction workers is inversely associated with the higher perception of risk and safety knowledge [51]. Training can reduce the rate of absenteeism and accidents, lower healthcare costs, and increase productivity [52]. Ergonomics training can improve employee knowledge of how they interact with the work environment as individuals and teams. This knowledge and the ability to apply it can decrease health-related issues and increase organizational performance. Several studies have shown that training significantly impacts overall job satisfaction and identifies work-related hazards [53,54]. Ergonomics training has previously been considered a key element in improving employee’s safety, well-being, and productivity [55,56]. All subscales of the ergonomics climate are essential, and it is beneficial to consider how they influence each other. Based on this study and previous studies, management commitment directly influences employee involvement, affecting the effectiveness of training and workers’ ability to identify and control hazards.

The mean ergonomics climate score was reported as significantly higher by employees who did not self-report pain than those who reported musculoskeletal pain. Hence, a higher perceived ergonomics climate is likely associated with lower musculoskeletal pain levels [57]. An ergonomics program that focuses on the design and modification of the workplace to improve overall health and operational performance can significantly impact employee perceptions regarding the ergonomics climate [8]. Implementing an effective ergonomics program can help reduce the prevalence of musculoskeletal disorders while also improving the efficiency and productivity of the employees [5,58,59]. A similar study also showed an association between psychosocial working conditions including low autonomy, low quality of leadership, and increased risk of reporting higher physical exertion [6].

A positive association was observed between the overall ergonomics climate and each of its subscales and organizational performance. Other studies have also observed a similar relationship. A positive correlation between applying ergonomics principles to reduce workplace-related problems and enhanced quality has been observed [60]. Additionally, the implementation of ergonomic and safety regulations to improve productivity and worker well-being can produce a more efficient production system [61,62]. Another study showed that enhancing efficiency and quality of work will reduce absenteeism and work-related injuries and diseases [63]. The implementation of ergonomics principles in designing a training program can be highly effective in individual’s learning performance [63]. Organizations that aim to improve their organizational performance should evaluate their ergonomics climate to develop targeted interventions [14].

Furthermore, the mean overall ergonomics climate score of employees with the absence of a disorder was significantly higher than the employees with the presence of disorder regarding their general health. The mean of each subscale score of ergonomics climate was higher in the employees with the absence of disorder than the group with the risk of developing the disorder. However, this difference was only significant in the management commitment and employee involvement subscales for operational performance and management commitment for employee well-being. Overall, higher levels of general health were reported when the organization had a higher ergonomics climate. It has been suggested that social supports of coworkers and supervisors in the workplace, as one of the characteristics of an ergonomics approach, can reduce the incidence rate of diseases in employees [64,65]. Similar to the significant relationship between each of the ergonomics climate subscales, including management commitment and employee involvement, was observed in this study, Vosoughi et al. [66] demonstrated that an organization’s climate and the relationships between employees and management had an impact on work-related stress. This stress had a positive correlation with the physical and mental well-being of employees [67]. Finally, other studies indicated that the organizational climate and management style could affect the mental well-being of employees [7,68,69]. Assessing the organization’s ergonomics climate and creating interventions based on the results could be an effective way to improve employees’ general health.

The present study was based on cross-sectional and self-reported data collected through three separate questionnaires. This type of study design and data have several limitations, such as the inability to determine a causal relationship and analyze data over time. The relationships between the ergonomics climate score and the outcomes are associations at the time of the questionnaire administration, and do not suggest causal inferences. Because the ergonomics climate measure is relatively new, and little research has been conducted using this measure, various outcomes were measured to investigate their possible association with the ergonomics climate scores. With many comparisons, this increases the possibility of getting a significant result simply by chance (type I error). Since this was the first study ever to use ergonomics climate to compare two occupational settings from the same industry the results should guide the path for future studies. Future studies should be conducted in various industries and workplaces, multilevel designs, and a large number of employees. Prospective cohort studies encompassing ergonomics climate measures would be beneficial in demonstrating possible causal pathways.

## 5. Conclusions

The translated (English to Persian) version of the Ergonomics Climate Assessment was an appropriate and beneficial tool for assessing the ergonomics climate’s status at two Iranian power plants. The ergonomics climate assessment can assist in prioritizing resources devoted to safety and production improvements within occupational settings, such as the power plants described in the present study. Furthermore, climate subscales can provide valuable and specific information to assist with targeted interventions for improving both worker well-being and operational metrics. The present study has demonstrated the cross-cultural use of the ergonomics climate assessment tool. A longitudinal study employing the ergonomics climate assessment tool to assess the effectiveness of systematic ergonomic interventions in a variety of occupational settings is needed.

This study confirms the findings of previously published work that implementing ergonomics’ principles focusing on improving both employee well-being and operational performance is beneficial for both the organization and employees. We believe that our findings have important policy implications. Future work policies should focus on leading indicators rather than lagging indicators to improve worker and organizational health and well-being [7]. Leading indicators allow organizations to be proactive rather than reactive in their safety and operational performance.

In the present study, higher ergonomics climate scores were associated with less self- reported musculoskeletal pain among the workers and higher operational performance for the company. Thus, businesses that focus their climate messages on the goals that include a system approach to workplace ergonomics will tend to have a healthier and more productive workforce.

## Figures and Tables

**Figure 1:**
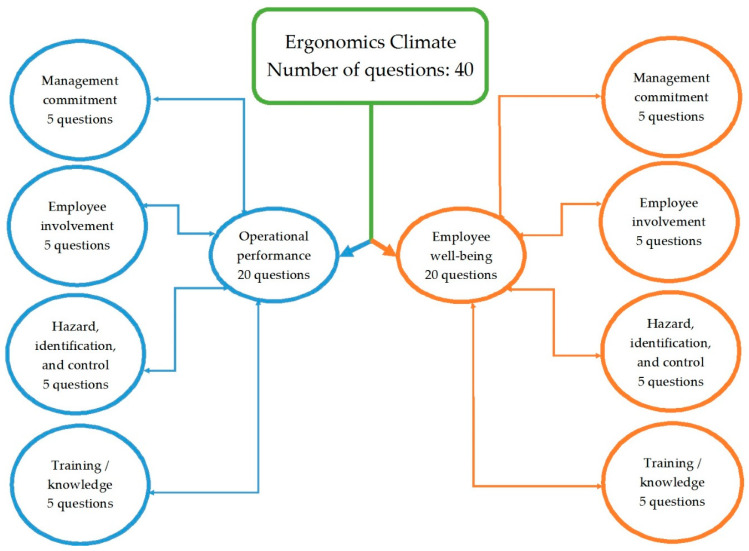
Ergonomics Climate Questions Structure.

**Table 1:** Additional information on the collected sample size.

	Total Numbers of Employee	Minimum Number of Required Sample	Number of Administrated Questionnaires with Consideration of 70% Response Rate	Received Valid Response
Besat power plant	290	106	150	109
Rey power plant	280	106	150	110

**Table 2:** Number of sampled employees sorted by departments.

	Power Plant Departments				
	Maintenance	Operation	Engineering	Planning and Administration	Total
Besat power plant	24	52	10	23	109
Rey power plant	36	45	11	18	110
Total	60	97	21	41	219

**Table 3:** Additional details for all three questionnaires.

Name	Developers	Number of Questions	Subscales	Scoring System	Reliability Coefficient	Ref.
Hersey and Goldsmith questionnaire	Hersey and Goldsmith, 1980	42	Ability (4 questions) Clarity (7 questions) Help (5 questions) Incentive (6 questions) Evaluation (9 questions) Validity (6 questions) Environment (5 questions)	Five-point Likert scale	*α* = 0.85	[35]
General health questionnaire	Goldberg and Hiller, 1979	28	Somatic symptoms (8 questions) Anxiety and insomnia (6 questions) Social dysfunction disorder (7 questions) Depression symptoms (7 questions)	The four-point scoring system using a binary method (0-0-1-1)	*α* = 0.90	[36,37]
Ergonomics climate assessment	Hoffmeister et al., 2015	40	Management commitment (10 questions) * Employee involvement (10 questions) * Job hazard identification and control (10 questions) * Training and knowledge (10 questions) *	Five-point Likert scale	*α* = 0.96	[30]

* Each subscale was assessed by two aspects of ergonomics climate (operational performance and employee well-being) as depicted in Figure 1.

**Table 4:** Demographic characteristics of sampled employees.

		Besat Power Plant		Rey Power Plant	
	Range	Frequency (N)	Frequency (%)	Frequency (N)	Frequency (%)
Age	<30	13	12	11	10
	30–34	45	41	57	52
	35–39	29	27	23	21
	40–44	8	7	4	3
	≥45	14	13	15	14
Gender	Female	5	4	1	1
	Male	104	95	109	99
Work experience (years)	<5	18	17	20	18
	5–10	58	53	60	55
	>10	33	30	30	27

**Table 5:** Comparison of the ergonomics climate sub-scales score between two power plants.

Ergonomics Climate Subscales	Besat	Rey	*p*-Value *
	(*N* = 108)	(*N* = 110)	
	Mean ± SD	Mean ± SD	
Operational performance	58.0 ± 19.1	70.8 ± 15.9	*p* < 0.001
Management commitment	15.0 ± 5.5	18.4 ± 4.2	*p* < 0.001
Employee involvement	14.2 ± 5.1	17.5 ± 4.5	*p* < 0.001
Hazard identification and control	15.2 ± 4.5	17.8 ± 4.0	*p* < 0.001
Training and knowledge	14.2 ± 5.0	17.0 ± 4.6	*p* < 0.001
Employee Well-being	55.8 ± 20.0	69.7 ± 16.9	*p* < 0.001
Management commitment	14.0 ± 6.0	18.0 ± 4.5	*p* < 0.001
Employee involvement	13.5 ± 5.3	17.2 ± 4.7	*p* < 0.001
Hazard identification and control	15.0 ± 4.8	17.6 ± 4.3	*p* < 0.001
Training and knowledge	13.8 ± 5.2	16.8 ± 4.7	*p* < 0.001
Overall ergonomics climate	113.7 ± 38.3	125.7 ± 31.7	*p* < 0.001

* Independent sample *t*-test.

**Table 6:** Comparison of ergonomics climate subscales between two employee groups regarding their general health condition.

Ergonomic Climate Subscales	General Health		*p*-Value *
	Absence of Disorder	Presence of Disorder	
	Mean ± SD	Mean ± SD	
Operational performance			
Management commitment	18.0 ± 4.5	16.2 ± 5.0	0.01
Employee involvement	16.7 ± 5.0	15.5 ± 4.8	0.01
Hazard identification and control	17 ± 4.5	16.3 ± 4.2	0.26
Training and knowledge	16.6 ± 5.1	15.3 ± 4.7	0.08
Employee Well-being			
Management commitment	17.5 ± 5.1	15.6 ± 5.5	0.01
Employee involvement	16.3 ± 5.4	14.9 ± 5	0.08
Hazard identification and control	16.7 ± 5.0	16.1 ± 4.4	0.4
Training and knowledge	16.0 ± 5.2	14.9 ± 4.9	0.12
Overall ergonomics climate	129.9 ± 32.9	116.7 ± 33.2	0.01

* Independent sample *t*-test.

## Data Availability

Not applicable.

## Appendix A

**Table A1:** Relationship between Ergonomics Climate sub-scales and self-reported Pain in a different part of the body.

		**Operational Performance**											
		**Management Commitment**			**Employee Involvement**			**Hazard Identification and Control**			**Training and Knowledge**		
**Self-Reported Pain**		**N**	**Mean ± SD**	***p*-Value ***	**N**	**Mean ± SD**	***p*-Value ***	**N**	**Mean ± SD**	***p*-Value ***	**N**	**Mean ± SD**	***p*-Value ***
Neck	No	168	16.7 ± 5.4	0.86	168	15.9 ± 5.2	0.73	166	16.5 ± 4.5	0.82	166	15.7 ± 5.1	0.72
	Yes	50	16.8 ± 4.2		50	15.7 ± 4.6		50	15.7 ± 4.2		50	15.5 ± 4.7	
Shoulder	No	185	16.8 ± 5.2	0.62	185	15.9 ± 5.3	0.79	183	16.5 ± 4.5	0.94	185	15.7 ± 5.0	0.72
	Yes	33	16.3 ± 4.5		33	15.7 ± 3.9		33	16.5 ± 4		33	15.4 ± 4.8	
Upper back	No	178	16.8 ± 5.3	0.76	176	16.0 ± 5.21	0.55	174	16.5 ± 4.6	0.68	174	15.8 ± 5.1	0.52
	Yes	42	16.5 ± 4.4		42	15.4 ± 4.6		42	16.8 ± 4.0		42	15.2 ± 4.6	
Elbow	No	206	16.7 ± 5.1	0.7	206	15.9 ± 5.1	0.54	204	16.5 ± 4.4	0.76	204	15.7 ± 4.9	0.6
	Yes	12	16.2 ± 5.8		12	15 ± 5.6		12	16.2 ± 6		12	14.7 ± 6.9	
Wrist	No	202	17.0 ± 4.9	0.008 **	202	16.1 ± 4.9	0.005 **	201	16.6 ± 4.4	0.71	201	15.8 ± 4.9	0.25
	Yes	16	13.4 ± 6.4		16	12.4 ± 6.0		15	16.1 ± 5.0		15	14.3 ± 5.9	
Lower back	No	178	17.1 ± 5.0	0.01 **	178	16.3 ± 5.0	0.01 **	177	16.7 ± 4.4	0.17	177	15.9 ± 5.0	0.15
	Yes	40	15.0 ± 5.2		40	14.2 ± 5.2		39	15.7 ± 4.4		39	14.6 ± 5.1	
Hip/Thigh	No	207	16.9 ± 5.0	0.008 **	207	16.1 ± 5.0	0.004 **	206	16.6 ± 4.4	0.11	206	15.8 ± 5.0	0.03 **
	Yes	11	12.3 ± 6.2		11	11.5 ± 5.7		10	14.3 ± 3.9		10	12.4 ± 3.0	
Knee	No	186	16.7 ± 5.0	0.08	186	16.1 ± 5.0	0.07	185	16.6 ± 4.5	0.55	185	15.8 ± 5.0	0.23
	Yes	32	15.3 ± 6.2		32	14.4 ± 5.7		31	16.0 ± 4.4		31	14.8 ± 5.1	
Ankle/Foot	No	201	16.9 ± 5.0	0.11	201	16.0 ± 5.0	0.27	200	15.8 ± 5.0	0.57	201	16.0 ± 5.0	0.18
	Yes	17	14.8 ± 6.2		17	14.6 ± 5.7		16	14.0 ± 4.7		17	14.6 ± 5.2	
		**Employee Well-Being**											
		**Management Commitment**			**Employee Involvement**			**Hazard Identification and Control**			**Training and Knowledge**		
**Self-Reported Pain**		**N**	**Mean ± SD**	***p*-Value ***	**N**	**Mean ± SD**	***p*-Value ***	**N**	**Mean ± SD**	***p*-Value ***	**N**	**Mean ± SD**	***p*-Value ***
Neck	No	168	16.3 ± 5.8	0.35	168	15.5 ± 5.4	0.6	166	16.4 ± 4.8	0.61	166	15.5 ± 5.3	0.65
	Yes	50	15.2 ± 5.2		50	15.0 ± 4.8		50	16.0 ± 4.5		50	15.1 ± 4.6	
Shoulder	No	185	15.5 ± 5.4	0.15	185	15.5 ± 5.4	0.41	183	16.4 ± 4.8	0.5	183	15.5 ± 5.3	0.48
	Yes	33	14.7 ± 4.4		33	14.7 ± 4.4		33	15.2 ± 4.5		33	14.8 ± 4.2	
Upper back	No	176	16.3 ± 5.7	0.26	176	15.5 ± 5.4	0.57	174	16.4 ± 4.8	0.45	174	15.5 ± 5.2	0.53
	Yes	42	15.2 ± 5.1		42	15.0 ± 4.7		42	15.8 ± 4.5		42	14.9 ± 4.7	
Elbow	No	206	16.2 ± 5.5	0.52	206	15.4 ± 5.3	0.97	204	16.4 ± 4.6	0.42	204	15.5 ± 5.0	0.23
	Yes	12	14.7 ± 7.9		12	15.3 ± 5.3		12	15.3 ± 6.0		12	13.7 ± 6.6	
Wrist	No	202	16.4 ± 5.4	0.009 **	202	15.6 ± 5.2	0.01 **	201	16.4 ± 4.7	0.19	201	15.6 ± 5.1	0.05 **
	Yes	16	12.6 ± 7.2		16	12.4 ± 5.8		15	14.8 ± 4.9		15	12.9 ± 5.5	
Lower back	No	178	16.6 ± 5.5	0.002 **	178	15.9 ± 5.2	0.05 **	177	16.7 ± 4.7	0.03 **	177	15.7 ± 5.1	0.02 **
	Yes	40	13.6 ± 5.8		40	13.3 ± 5.2		39	14.8 ± 4.4		39	13.7 ± 5.0	
Hip/Thigh	No	207	16.3 ± 5.5	0.04 **	207	15.6 ± 5.2	0.007	206	16.4 ± 4.7	0.24	206	15.5 ± 5.1	0.05 **
	Yes	11	12.8 ± 7.9		11	11.2 ± 4.8		10	14.6 ± 4.4		10	12.3 ± 5.2	
Knee	No	186	16.4 ± 5.5	0.09	186	15.7 ± 5.2	0.05 **	185	16.5 ± 4.8	0.27	185	15.6 ± 5.1	0.1 **
	Yes	32	14.5 ± 6.6		32	13.8 ± 5.8		31	15.5 ± 4.5		31	14 ± 5.4	
Ankle/Foot	No	201	16.3 ± 5.5	0.02 **	201	15.6 ± 5.2	0.07	200	16.5 ± 4.7	0.2	200	15.5 ± 5.1	0.07
	Yes	17	13.1 ± 7.1		17	13.2 ± 6.0		16	14.6 ± 4.7		16	13.2 ± 4.8	
**Overall Ergonomics Climate**													
**Self-Reported Pain**		**N**	**Mean ± SD**	***p*-Value ***									
Neck	No	168	121.27 ± 36.90	0.25									
	Yes	50	114.72 ± 30.33										
Shoulder	No	185	120.69 ± 36.34	0.36									
	Yes	33	114.60 ± 30.61										
Upper back	No	176	120.75 ± 35.99	0.40									
	Yes	42	115.66 ± 33.68										
Elbow	No	206	120.62 ± 35.38	0.14									
	Yes	12	105.25 ± 36.60										
Wrist	No	202	121.02 ± 34.90	0.045 **									
	Yes	16	104 ± 40.81										
Lower back	No	178	122.68 ± 34.88	0.01 **									
	Yes	40	106.82 ± 3.97										
Hip/Thigh	No	207	120.90 ± 34.83	0.04 **									
	Yes	11	98.45 ± 43.45										
Knee	No	186	121.55 ± 35.47	0.07									
	Yes	32	109.43 ± 34.67										
Ankle/Foot	No	201	121.25 ± 35.34	0.03 **									
	Yes	17	102.29 ± 34.15										

* Independent sample *t*-test. ** *p* < 0.5.
